# Amnesia Associated with Bilateral Hippocampal and Bilateral Basal Ganglia Lesions in Anoxia with Stimulant Use

**DOI:** 10.3389/fneur.2017.00027

**Published:** 2017-02-08

**Authors:** Marc W. Haut, Jeffery P. Hogg, Patrick J. Marshalek, Blair C. Suter, Liv E. Miller

**Affiliations:** ^1^Department of Behavioral Medicine and Psychiatry, West Virginia University School of Medicine, Morgantown, WV, USA; ^2^Department of Neurology, West Virginia University School of Medicine, Morgantown, WV, USA; ^3^Department of Radiology, West Virginia University School of Medicine, Morgantown, WV, USA; ^4^West Virginia University School of Medicine, Morgantown, WV, USA

**Keywords:** amnesia, basal ganglia, hippocampus, stimulant, anoxia

## Abstract

We report a case of a 55-year-old man with ischemic lesions of the bilateral hippocampus and bilateral basal ganglia following a myocardial infarction during an episode of multiple drug use with subsequent anoxia requiring resuscitation. He presented for a neuropsychological evaluation with an anterograde amnesia for both explicit and procedural memory. There are two main points to this case, the unique aspects of the bilateral multifocal lesions and the functional, cognitive impact of these lesions. We hypothesize that his rare focal bilateral lesions of both the hippocampus and basal ganglia are a result of anoxia acting in synergy with his stimulant drug use (cocaine and/or 3,4-methylenedioxy-methamphetamine). Second, his unique lesions produced an explicit and implicit/procedural anterograde amnesia.

## Introduction

A 55-year-old right-handed Caucasian man presented for a neuropsychological evaluation on referral from a family member. He was in a residential treatment facility for drug abuse. Family members became concerned that the patient was not learning new information and requested a neuropsychological evaluation. For example, he was having trouble learning what had happened to him that lead to his hospitalization and then rehabilitation treatment. Family indicated that he has trouble recalling what they had discussed 5 min earlier.

History of the episode was obtained from the patient, his brother, and review of available medical records. He had spent a weekend using substances with a woman in a hotel. At some point, the patient became unresponsive. He was resuscitated by the woman who then left for fear of arrest. He was eventually taken to a local hospital.

EKG at the time of admission showed normal sinus rhythm, but a prolonged QTC of 501, which was lengthened compared to an examination 1.5 years before. His blood pressure on admission was 169/89.

His troponin level was elevated (2.21, reference range of <0.05 ng/mL). He had a cardiac catheterization that showed a left ventricular ejection fraction of 50% with mild hypokinesis of the apex, normal end diastolic pressure of 10 mmHg, normal left main coronary artery, minimal lesion of the left anterior descending artery, normal left circumflex artery, and 50% distal lesion in the right coronary artery. He was diagnosed with a non-ST segment elevation myocardial infarction (MI).

On admission, urine drug screen was positive for opiates, cocaine, and amphetamines and negative for barbiturates, benzodiazepines, methadone, phencyclidine, and tetrahydrocannabinol. Liver function tests were abnormal: AST = 115 (reference range: 15–37 U/L), ALT = 342 (reference range: 12–78 U/L) but returned to the normal range within 3 weeks (AST = 16, reference range: 10–35 U/L; ALT = 25, reference range: 9–46 U/L).

**Figure 1:**
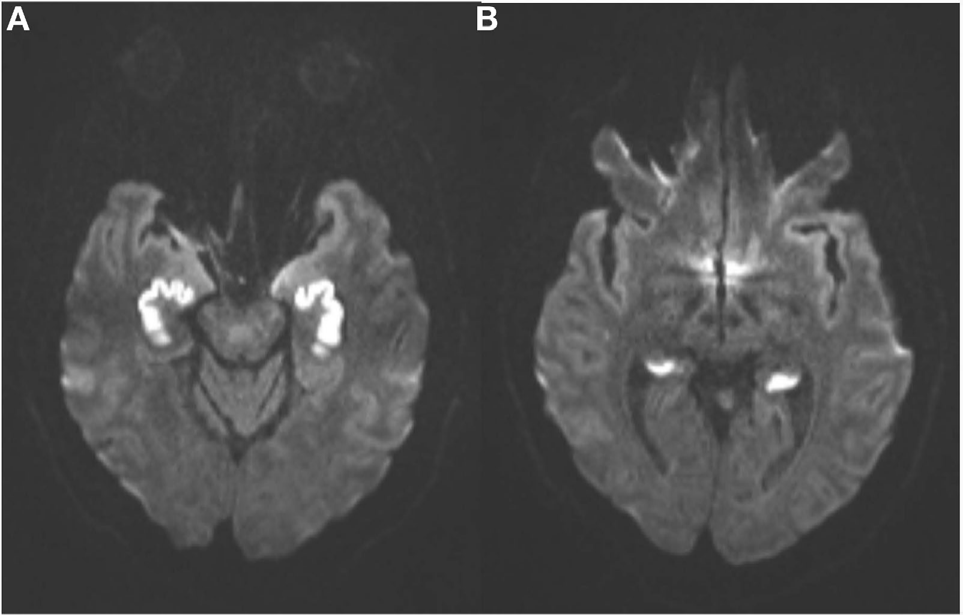
**(Axial diffusion-weighted imaging): symmetrical abnormal hyperintense signal in the anterior (A) and posterior (B) hippocampal formations indicate irreversible cytotoxic edema from infarction**.

**Figure 2:**
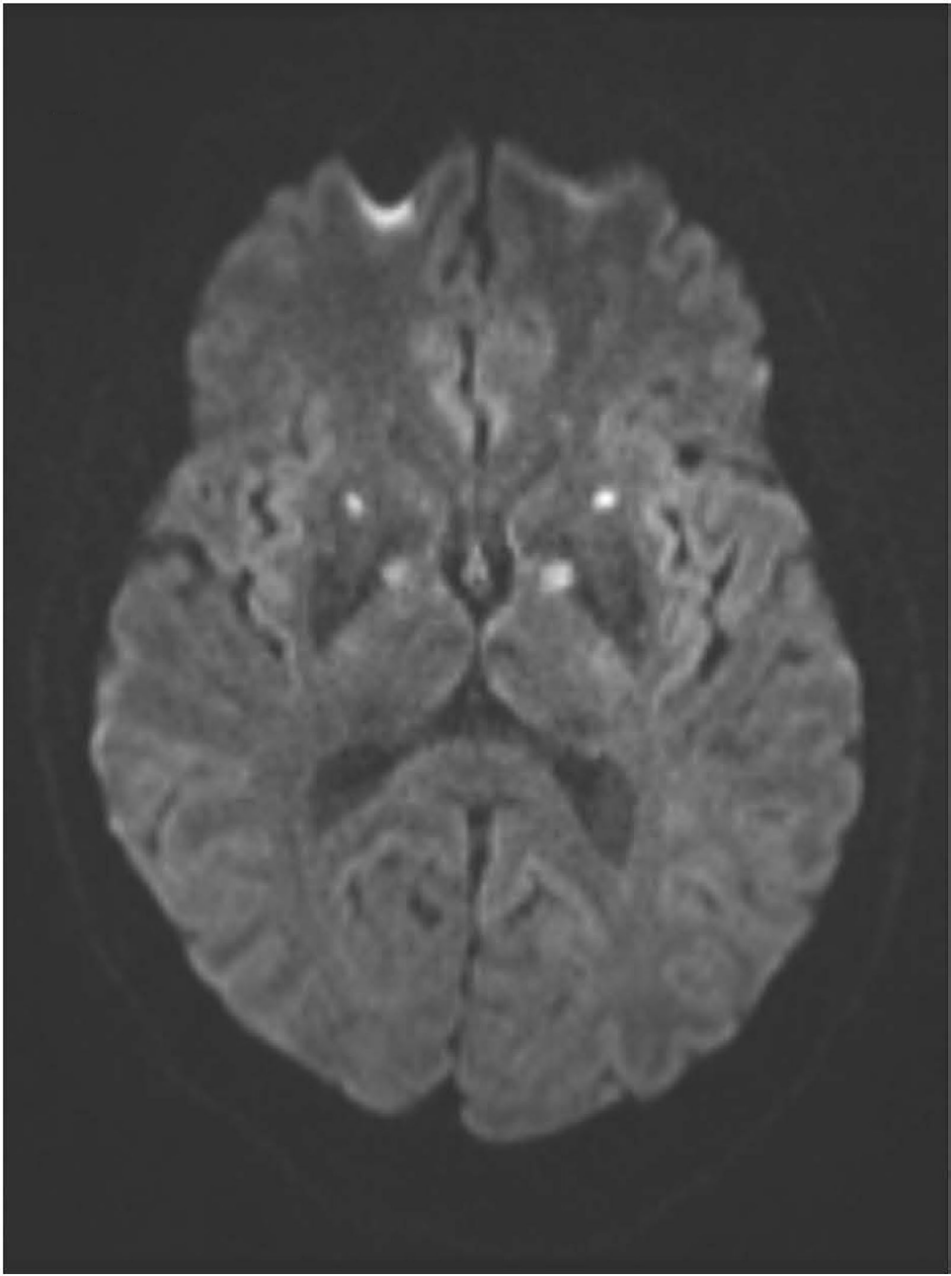
**(Axial diffusion-weighted imaging): infarctions in the globi pallidi and anterior putamina are indicated by similar symmetrical foci of abnormal hyperintense signal**.

**Figure 3:**
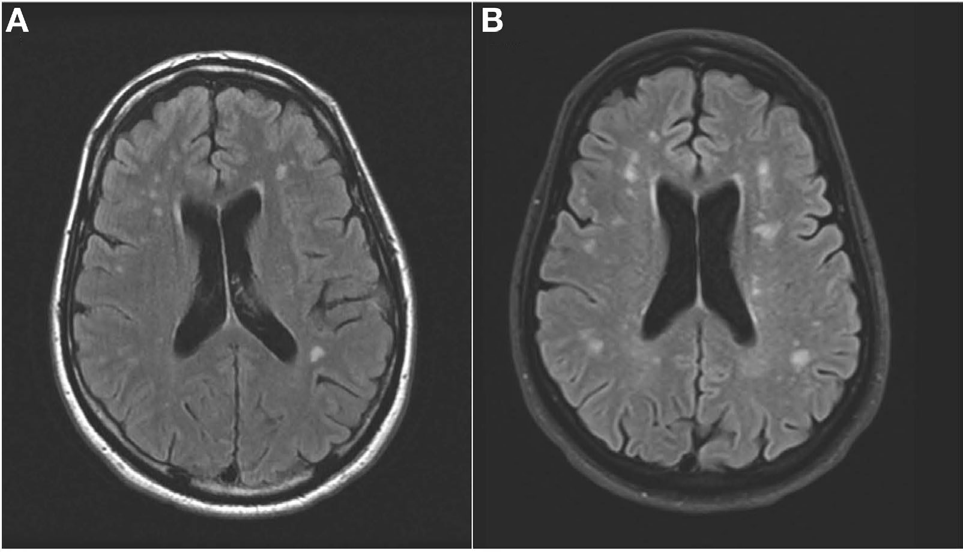
**(Axial FLAIR): hyperintense signal in the periventricular white matter that increased from prior to the event (A) to the time of the acute event (B)**.

An MRI scan of the brain on admission showed acute ischemic lesions on diffusion-weighted imaging affecting the entire length of hippocampus bilaterally (see Figures 1A,B) as well as the bilateral basal ganglia including the globus pallidus and the anterior putamen (see Figure 2). Susceptibility weighted imaging showed no areas of hemorrhage. T2/FLAIR hyperintensities suggesting chronic microvascular ischemic disease in the subcortical and periventricular white matter were present and mildly increased in the admission brain MRI compared to a prior brain MRI done 2 months earlier for headache complaints. However, that prior study did not have restricted diffusion to indicate acute ischemia in the hippocampus or basal ganglia (see Figures 3A,B). Computed tomographic angiography at the time of the event did not reveal any significant stenosis but showed evidence of the small vessel ischemic changes. After stabilization, he was discharged and then admitted to a residential treatment center for alcohol and drug abuse.

Other history includes long-standing substance abuse including alcohol, 3,4-methylenedioxy-methamphetamine (MDMA), and marijuana beginning in high school. He had been using MDMA twice a week for the last 2 years and alcohol Thursdays through Sundays. He had a college education, was a Certified public accountant, and owned an accounting firm. He had medication-controlled hypertension and a 15 pack a year smoking history for 30 years with chronic obstructive pulmonary disease (COPD). He took hydrocodone–acetaminophen 5–325 every 6 h for chronic pain, which would explain the opiates in the urine drug screen.

Neuropsychological evaluation 10 weeks after this acute event revealed average intelligence [Wechsler Adult Intelligence Scale-IV (1) Full Scale IQ = 92]. He showed an inability to learn new explicit information on multiple standard clinical tests of memory. On the California Verbal Learning Test II (CVLT-II) (2), he had a reduced learning curve and was amnestic at a delay and chance on recognition. He also had 11 intrusion errors (recalling words that were not on the list) during his free recall. On the Logical Memory and Visual Reproduction subtests of the Wechsler Memory Scale Revised (3), he was amnestic at the delays for both these measures. We also administered an experimental measure of procedural memory. For this task we used the Trail Making Test, part A (4). This task requires individuals to use a pencil to connect a series of numbers scattered about a page in order (1–25) as quickly as possible. Over five trials, as expected, his time decreased from 30 s (*T*-score = 46, mean = 50, SD = 10) to 15 s, indicating that he learned the locations of the numbers. However, after a 30-min delay his time to complete the task increased almost back to his original time to 24 s suggesting that he did not fully consolidate and retain this new procedural learning.

## Background

There are two main points to this case, the unique aspects of the bilateral multifocal lesions and the functional, cognitive impact of those lesions. Bilateral ischemic lesions of the hippocampus can occur by occlusion, which is reported, but rare (5). More commonly, bilateral lesions of the hippocampus occur from anoxia as the CA1 neurons are particularly vulnerable (5). In addition, bilateral lesions of the hippocampus have occurred in the context of cocaine use as the result of vasospasm without anoxia (6, 7). We are aware of two cases of bilateral involvement of the hippocampus and bilateral involvement of the basal ganglia in anoxia and stimulant use. We have also seen another case of bilateral hippocampal and basal ganglia lesions in the setting of anoxia complicated by multidrug use including stimulants (8). In that case, the individual was found down and had been using “bath salts,” which typically include stimulants. That prior case also had evidence of an anterograde amnesia for explicit memory and procedural memory. There is an additional case in the literature with bilateral hippocampal and bilateral basal ganglia lesions (9). That case involved anoxia after cocaine-induced stroke of the hippocampus. The patient was found unresponsive and also had an anterograde amnesia.

One of the unique aspects of our case is the type of amnesia this patient demonstrated, for both explicit and implicit/procedural, which we believe is a function of having bilateral hippocampal and bilateral basal ganglia lesions. To support our hypothesis, we compare the performance of the current case to other cases of amnesia that showed intact performance on our measure of implicit/procedural memory. For example, a 70-year-old right-handed woman in the early stages of Alzheimer’s disease had a failure to consolidate new explicit memory on the CVLT. However, she showed ability to learn and retain new procedural motor information on the Trail Making Test, Part A with initial time to complete of 37 s (*T*-score = 50). Her time to complete the task decreased to 20 s over five trials and remained at 23 s over a 30-min delay period, indicating that she retained most of the motor learning over time. We have also seen a 60-year-old right-handed male with Wernicke–Korsakoff’s amnesia. He was amnestic on standard clinical measures but showed learning and retention on the modified Trails A. Specifically, initial time to complete was 110 s (*T*-score = 16). After five trials he improved to 88 s and then was at 80 s after a delay. Finally, we can examine performance on modified Trails A to a 67-year-old woman who had complaints of memory problems but had normal performance on neuropsychological evaluation. This woman initially took 33 s to complete Trails A (*T*-score = 53) and after five trials took 16 s to complete the task. After a 30-min delay, she completed the task in 19 s, thus maintaining most of the motor learning over time. Thus, we have two other cases of amnesia for explicit material and a normal individual who could learn and retain on modified Trail A measure of procedural memory, but the present case did not show that retention of procedural learning.

## Discussion

We present a patient with an anoxic event in the context of drug use who demonstrated clinical evidence of an anterograde amnesia for both explicit and implicit/procedural information. We will discuss the possible etiological factors in the production of the relatively unique bilateral hippocampal and bilateral basal ganglia lesions as well as the evidence of the neuroanatomical correlates with his amnesia.

Anoxia is well known to cause ischemic lesions of the hippocampus (10) and can produce severe anterograde amnesia. Less commonly, bilateral lesions of the basal ganglia have been reported in anoxia (11, 12). Other causes of bilateral basal ganglia lesions include carbon monoxide exposure (13, 14). In this case, we hypothesize that the anoxia secondary to MI and/or respiratory arrest during drug overdose, produced the bilateral lesions of the hippocampus. The addition of drug use at the time of the cardio/respiratory arrest, specifically stimulants, exacerbated the impact of the anoxia, producing additional lesions in the basal ganglia. Stimulants specifically prime the basal ganglia for anoxic injury by making the tissue more susceptible to reduced glucose and oxygen, and this is hypothesized to be a function of either mitochondrial dysfunction or a persistent depolarization of dopamine terminals (15). Stimulants, specifically cocaine, which was in his system, can produce ischemic lesions from vasospasm. There are prior cases of bilateral basal ganglia lesions in stimulant use. For example, there is a case of bilateral basal ganglia lesions following MDMA use (16), the drug of choice for our patient, as well as an additional case involving methadone, cocaine, and amphetamines (17) producing basal ganglia lesions. A combination of cocaine and alcohol resulting in unconsciousness has also produced bilateral basal ganglia lesions (18). There was an additional case of bilateral basal ganglia lesions with methadone and benzodiazepines (19), but as can be seen from the above cases, the majority involved a stimulating drug. Finally, an autopsy series of consecutive cases of bilateral globus pallidus necrosis found that 10 of 27 cases involved drug overdose, more than any other etiology (20). However, none of the above cases, except for our previous case (8), noted the combination of bilateral hippocampal and basal ganglia lesions. There is a case in the literature with bilateral hippocampal and bilateral basal ganglia lesions (9). That case involved anoxia after cocaine-induced ischemic stroke of the hippocampus. The patient was found unresponsive, and it was theorized that increased dopamine produced vasospasm and reduced blood flow. The imaging in that case clearly showed bilateral basal ganglia lesions, in addition. Note is made that dopamine-rich regions are likely targets, which would include the basal ganglia. When considering the animal literature (21, 22), it is possible that a combination of excitotoxicity and oxidative stress at the level of the central nervous system, and general metabolic compromise at the systemic level played a role in the development of the lesions. However, we hypothesize increased dopamine from stimulant use may prime injury to the basal ganglia and hippocampus during anoxia by producing vasospasms in those specific regions of the brain.

An additional factor to consider is the role of cigarette smoking, stimulant use, carbon monoxide levels, and COPD. Our patient has a 15 pack-year history of cigarette smoking and an established diagnosis of COPD. It could be that preexisting COPD-producing hypoxemia altered the presentation of acute anoxia. COPD can certainly impact brain structure and cognition, independent of smoking history (23). Stimulant users are known to smoke cigarettes at higher rates. This is not surprising as co-occurring stimulant use and cigarette smoking is about 90% (24). Heavy smokers are noted to have elevated carboxyhemoglobin levels (25). Of course, the interaction of carbon monoxide, anoxia, preexisting COPD, and stimulant use may explain the unique presentation in this case.

We hypothesize that the unique lesions of this patient produced his amnesia for explicit and procedural information. It is hypothesized the bilateral hippocampal lesions produced the explicit amnesia and the bilateral basal ganglia lesions produced the implicit/procedural amnesia. Performance on standard clinical measures of explicit memory indicated an inability to learn and consolidate new information. Not only was he unable to recall or recognize new information after a delay but also he made intrusion errors on free recall (recalling words that were not presented). This type of error indicates problems with consolidation, which is typically attributed to the hippocampus. Performance on our experimental measure of procedural learning also indicated a failure to consolidate new information based on the loss of the improvement in time to complete the task over a delay period.

When we compare our current case with a patient with Alzheimer’s disease, the essential neuroanatomical difference between these two cases is involvement of the basal ganglia. Our current case and the patient with Alzheimer’s disease both have damage to the hippocampus, which produces classic anterograde amnesia for explicit information. However, only the current case has focal involvement of the bilateral basal ganglia in addition to the bilateral hippocampal lesions. Prior cases have shown that lesions limited to the hippocampus have intact implicit memory (10). Other cases of amnesia involving the hippocampus also show impaired explicit memory with intact procedural learning and retention. For example, the famous case of HM had intact learning and retention on a mirror tracing task, a well-established experimental measure of procedural learning (26). In addition, intact procedural learning has been reported in cases of Herpes Encephalitis, which involves the hippocampus, when explicit memory is impaired (27, 28).

We found in our current case that he did not maintain the improvement he demonstrated in time to completion over the delay on the modified Trail Making Test to the same degree as a patient with Alzheimer’s disease, a patient with a Korsakoff’s amnesia and a normal individual, suggesting impairment of procedural learning and retention. Our finding of impairment with procedural learning and retention is consistent with the known literature that procedural learning involves the basal ganglia. In a classic study, patients with Huntington’s disease and Alzheimer’s disease showed different impairment on a procedural/motor learning task (29). Patients with Alzheimer’s disease showed intact procedural learning, while patients with Huntington’s disease showed impaired procedural learning. In addition, the cases discussed above of intact procedural memory when the basal ganglia are not involved provide a double dissociation. We used an experimental procedure for clinically assessing procedural learning. This case shows the potential clinical value of using the Trail Making Test as a measure of procedural learning, but more study of this measure is required including performance of a group of normal controls as well as different patient groups such as patients with Alzheimer’s disease, Parkinson’s disease, and Huntington’s disease.

## Concluding Remarks

Anoxia is well known to produce ischemic hippocampal lesions, but less so basal ganglia lesions. The addition of stimulants, such as cocaine or other stimulants such as MDMA, at the time of an anoxic event may produce isolated bilateral lesions involving both the hippocampus and basal ganglia from vasospasm. We emphasize that our conclusions are tentative as it is based on this single case and two other cases in the literature. A case controlled study would be required to draw stronger conclusions about the additive role stimulants may play in producing discrete lesions in the context of anoxia. Clinically, if isolated bilateral lesions of both the hippocampus and basal ganglia occur, learning and memory can be affected for both explicit and implicit/procedural learning. This type of amnesia may have a particularly negative impact on functional outcome.

## Ethics Statement

The study was approved by the West Virginia University Institutional Review Board. After review, this case report was declared exempt from patient consent.

## Author Contributions

MH: data acquisition, analysis, and interpretation; drafting intellectual content; final approval; and responsibility for content. JH: data analysis and interpretation; drafting intellectual content; final approval; and responsibility for content. PM: data interpretation; drafting intellectual content; final approval; and responsibility for content. BS: data interpretation; drafting intellectual content; final approval; and responsibility for content. LM: data acquisition and interpretation; drafting intellectual content; final approval; and responsibility for content.

## Conflict of Interest Statement

The authors declare that the research was conducted in the absence of any commercial or financial relationships that could be construed as a potential conflict of interest.
